# Alzheimer’s Disease from the Amyloidogenic Theory to the Puzzling Crossroads between Vascular, Metabolic and Energetic Maladaptive Plasticity

**DOI:** 10.3390/biomedicines11030861

**Published:** 2023-03-11

**Authors:** Michele Cerasuolo, Michele Papa, Anna Maria Colangelo, Maria Rosaria Rizzo

**Affiliations:** 1 Department of Advanced Medical and Surgical Sciences, University of Campania “Luigi Vanvitelli”, 80138 Naples, Italy; 2 Laboratory of Neuronal Networks Morphology and System Biology, Department of Mental and Physical Health and Preventive Medicine, University of Campania “Luigi Vanvitelli”, 80138 Naples, Italy; 3 SYSBIO Centre of Systems Biology ISBE-IT, 20126 Milan, Italy; 4 Laboratory of Neuroscience “R. Levi-Montalcini”, Department of Biotechnology and Biosciences, NeuroMI Milan Center for Neuroscience, University of Milano-Bicocca, 20126 Milano, Italy

**Keywords:** Alzheimer’s disease, neurovascular unit, blood–brain barrier, glia, reactive oxygen species (ROS)

## Abstract

Alzheimer’s disease (AD) is a progressive and degenerative disease producing the most common type of dementia worldwide. The main pathogenetic hypothesis in recent decades has been the well-known amyloidogenic hypothesis based on the involvement of two proteins in AD pathogenesis: amyloid β (Aβ) and tau. Amyloid deposition reported in all AD patients is nowadays considered an independent risk factor for cognitive decline. Vascular damage and blood–brain barrier (BBB) failure in AD is considered a pivotal mechanism for brain injury, with increased deposition of both immunoglobulins and fibrin. Furthermore, BBB dysfunction could be an early sign of cognitive decline and the early stages of clinical AD. Vascular damage generates hypoperfusion and relative hypoxia in areas with high energy demand. Long-term hypoxia and the accumulation within the brain parenchyma of neurotoxic molecules could be seeds of a self-sustaining pathological progression. Cellular dysfunction comprises all the elements of the neurovascular unit (NVU) and neuronal loss, which could be the result of energy failure and mitochondrial impairment. Brain glucose metabolism is compromised, showing a specific region distribution. This energy deficit worsens throughout aging. Mild cognitive impairment has been reported to be associated with a glucose deficit in the entorhinal cortex and in the parietal lobes. The current aim is to understand the complex interactions between amyloid β (Aβ) and tau and elements of the BBB and NVU in the brain. This new approach aimed at the study of metabolic mechanisms and energy insufficiency due to mitochondrial impairment would allow us to define therapies aimed at predicting and slowing down the progression of AD.

## 1. Introduction

Alzheimer’s disease (AD) is a progressive and degenerative disease that affects cognition and leads to the most common type of dementia worldwide. The number of people living with dementia—estimated to stand at 55 million in 2019—is expected to rise to 139 million in 2050, considering the overwhelming rate of estimated diagnosis (one every three seconds) and the increased lifespan [1]. AD was described in the first decade of the twentieth century by Alois Alzheimer and Gaetano Perusini [2]. According to the amyloid hypothesis, two proteins are mainly involved in AD pathogenesis: amyloid β (Aβ) and tau [3]. Aβ length, polymerization, and conformation seem to be the main factors responsible for the formation of fibrils and plaques [4]. The amyloid plaques apparently cannot be processed by the resident scavenger cells, thus Aβ accumulates in the extracellular matrix (ECM) and impairs cell function. Hyperphosphorylated tau, instead, is responsible for intracellular neurofibrillary tangles which affect microtubule assembly, sequester microtubule-associated proteins (MAPs), and potentially block the multiple MAPs assembly, responsible for vesicular trafficking and sorting in neurons [5,6]. Clinical, functional, and histopathological evidence supports that the medial temporal lobe, including the entorhinal cortex and hippocampus, is the most vulnerable region to AD pathophysiology or, at least, where neuronal dysfunction and functional disconnection occur more prominently [7]. The localization of these plaques and neurofibrillary tangles follows a constant pattern: they accumulate initially in the entorhinal cortex, proceed towards the limbic and hippocampal structures, and then spread to the frontal, temporal, and parietal neocortex [8]. The amyloid hypothesis in AD pathogenesis has proved disappointing, considering the high expectations for the first Aβ immunization (suspended due to the severe encephalitis in some participants). The postmortem evaluation of treated patients demonstrated a complete amyloid removal (the immunotherapy was indeed effective), but there were no differences in terms of both survival and disease progression comparing the immunized to the untreated group [9]. Disease-modifying drugs targeting Aβ (both monoclonal antibodies and γ-secretase inhibitors) failed to show benefits in phase III clinical trials [10]. Aducanumab is the only antibody against Aβ currently submitted to the FDA for marketing approval, but there are still questions about its efficacy [11]. Following the failure of therapeutic strategies aimed at countering beta-amyloid plaques, there has been a great impulse to move from targeting amyloid to tau. Tau is a microtubule-associated protein found primarily in central nervous system (CNS) neurons, although it is also expressed at low levels in astrocytes and oligodendrocytes [12]. It has been shown in imaging-based and autopsy studies to correlate more closely with the cognitive decline of Aβ [13]. Immunotherapy targeting tau is in development and has entered phase II trials. However, no evidence shows that MBAs will enter neurons to bind and clear abnormal or toxic forms of tau. It is also unclear whether antibodies targeting tau capture and move pathological forms from the brain to the periphery, neutralize toxic forms of tau protein in situ, or stimulate phagocytosis through microglia. Therefore, the therapeutic mechanisms through which the accumulation of pathological forms of tau protein could be acted on are still unclear [12].

**Figure 1:**
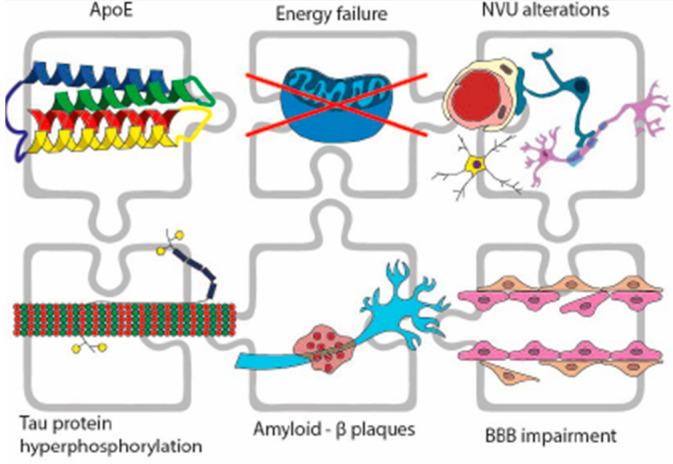
Genetic, metabolic, vascular, and energetic factors contribute to the onset and progression of Alzheimer’s disease.

Moreover, amyloid deposition around cerebral vessels is related to cerebral amyloid angiopathy (CAA), which is observed almost in all AD patients to different degrees [14], and is considered, even after controlling for age and AD pathology, an independent risk factor for cognitive decline [15,16]. CAA is a major contributor to vascular damage and BBB failure in AD; indeed, BBB leakage was evaluated as a mechanism for CAA-related brain injury with increased deposition of both immunoglobulins and fibrin [17]. Furthermore, the BBB dysfunction has been considered an early biomarker of cognitive decline and early stages of clinical AD [18,19,20]. It has been shown that carriers of apolipoprotein E4 (*APOE4*) (ε3/ε4 or ε4/ε4), an identified genetic risk factor for AD, show a higher BBB permeability in the medial temporal lobe and hippocampus compared with non-carriers, even when cognitively healthy [21]. The BBB breakdown was more severe in carriers with cognitive impairment but was not related to AD biomarkers (both β-amyloid and tau). The BBB damage, measured in vivo, considering pericytes and platelet-derived biomarkers predicted the future cognitive status in carriers, even after controlling the analysis for Aβ and tau levels [21]. These predictive biomarkers correlated with increased cyclophilin A (CypA)–matrix metalloproteinase-9 (MMP9) activity in the cerebrospinal fluid. *APOE* regulates neurodegeneration predominantly by modulating activation of microglia, although a minor role for *apoE* in regulating the formation of tau and insoluble tau has also been identified regarding immunomodulatory function. Ptau (Ser202 and Thr205 epitopes) progression is therefore also determined by microglia [22], just as there is strong evidence that reactive oxygen species (ROS) directly promote tau modifications [23]. Vascular damage generates hypoperfusion and relative hypoxia in areas with high energy demand. Aβ, as recently demonstrated, could also directly narrow brain capillaries at pericyte sites [24]. Aβ activates ROS formation, which prompts the release of endothelin-1 (ET-1) and mediates capillary constriction [24]. Long-term hypoxia and the accumulation within the brain parenchyma of neurotoxic molecules could be seeds of a self-sustaining pathological process [25,26]. Cellular dysfunction comprises all the elements of the neurovascular unit (NVU) [26]. Neuronal loss seems to be consistently related to energy failure and mitochondrial impairment [27]. The cerebral glucose metabolism seems to be progressively affected and shows a specific regional distribution. This energy deficit worsens throughout aging and can be observed in the pre-clinical stages of neurodegenerative disorders of aging (NDAs) [28,29]. Even before the diagnosis of AD, there is a disruption of glucose metabolism in some regions, but oxygen, lactate, and ketone levels do not change significantly. In clinically significant mild cognitive impairment (MCI), there is a deficit of glucose uptake of 10–12% in the entorhinal cortex and in the parietal lobes, a defect that becomes more accentuated as the disease advances [27]. Understanding the complex interactions between β amyloid (Aβ) and tau and elements of the NVU in the central nervous system can contribute to the development of new therapies aimed at predicting and slowing the progression of AD. Therefore, here we describe the new perspective analyzing the interactions within the NVU elements of the BBB with the related metabolic consequences, focusing on energy failure and mitochondrial impairment (Figure 1).

## 2. Vascular Damage: Neurovascular Unit, BBB Disruption, and Pathological Blockage of the Glymphatic Flow

The neurovascular unit (NVU) manages the metabolic supply of the brain [30]. The NVU is finely regulated to support the regional energy demand of neurons and glia through the blood–brain barrier (BBB). Moreover, it allows the clearing of cellular and extracellular by-products (e.g., CO_2_, lactate, Aβ, tau) [31]. The intimate anatomical and chemical relationship between NVU elements prompts delivery of oxygen and glucose in selected cerebral areas synchronously with their activation. The mechanisms regulating vasodilation and vasoconstriction have been described both as feedback (metabolic demand and waste clearance drive vasodilation) and feedforward (release of vasoactive substance through synaptic activity, independent of the metabolic needs), and are not mutually exclusive [31]. The cerebral blood flow in resting conditions and the vascular responses to activation are impaired in early phases of AD, suggesting prodromal changes of these mechanisms during the disease [32]. The NVU of AD brains is damaged particularly at the microvascular level with injured and rarefied capillaries showing a thickened basement membrane [33]. Brain atherosclerosis, ischemic lesions, and CAA are more pronounced in the AD population compared to aged-matched controls [34,35,36]; however, BBB damage has been shown as an early phenomenon in patients with MCI due to AD [37]. Degeneration of pericytes, measured by analyzing in vivo markers, seems to be crucial for BBB leakage [37]. They play a crucial role supporting the structural integrity and genesis of the BBB [38]. The maintenance of vascular integrity in the brain is also regulated by the communication between astrocyte end-feet and pericytes [39]. Pericytes (PCs) are the cells of microvessels including capillaries, venules, and arterioles that wrap around the endothelial cells. They provide structural support and dynamic capacity to the microvasculature. There is an accelerated degeneration of pericytes associated with BBB breakdown in AD brains with *ApoE4* carriers, and E4 fails to suppress the CypA-MMP-9 pathway in pericytes leading to degradation of BBB tight junctions [40]. Dysfunction of the pericytes due to the accumulation of Aβ plaques also leads to cerebral hypoperfusion which further worsens the clinical picture [41]. In the human brain, *APOE* and the nuclear factor of activated T cells (*NFAT*) are selectively dysregulated in pericytes of *APOE4* carriers, and inhibition of calcineurin–NFAT signaling reduces *APOE4*-associated CAA pathology in vitro and in vivo. Considering the role of pericytes in *APOE4*-mediated CAA calcineurin–*NFAT* signaling could be a therapeutic target in CAA and Alzheimer’s disease [42]. The early involvement of pericytes could characterize these cells as more sensitive to the metabolic impairment. Vascular damage could be considered the initial input through which impaired BBB function and/or reduced cerebral perfusion determines secondary neuronal damage, followed by Aβ deposition, functional impairment, and gray matter atrophy [43]. Vascular dysfunction can be triggered by degeneration and loss of pericytes and the decrease in cerebral blood flow in patients with AD. Pericyte denaturation could be responsible for an early reduction in O_2_ supply in the active sites of the brain [32]. LRP-1 is a member of the low-density lipoprotein receptor family, while RAGE is a multiligand receptor of the immunoglobulin superfamily. They are mainly located on the surface of endothelial cells and pericytes [38]. Several studies have shown that by regulating the levels of LRP-1 and RAGE, it is possible to modulate the transport and clearance of β-amyloid plaques [44,45]. BBB-associated pericytes regulate the clearance of Aβ aggregates via an LRP1/apoE isoform-specific mechanism, with *apoE4* disrupting Aβ clearance compared to *apoE3*. This has been demonstrated by pharmacologic inhibition of LRP1 by an anti-LRP1 antibody. *ApoE* isoforms can be considered either protective (*ApoE2*) or a major risk for AD development (*ApoE4*), and, interestingly, *ApoE4* homozygotes display, during asymptomatic stages even decades before the AD onset, a similar regional hypometabolism compared to AD patients, as observed with glucose tracers [46]. The precise understanding of how the *ApoE4* isoform differently interacts with the metabolic receptor LRP1 seems a pivotal step in BBB regulation and AD pathophysiology [47]. A possible therapeutic target to control Aβ levels and clearance could therefore be represented by the LRP1/apoE pathway in pericytes [48]. The formation and maintenance of the BBB are influenced by the presence of astrocytic end-feet interacting with the endothelium and pericytes, capable of building up this delicate and complex framework even in vitro [49]. Astrocytes are cells characterized by dense branched processes that extend both into the synaptic cleft and towards the blood vessels. The astrocytes are highly heterogeneous through the CNS and form functional domains, particularly through gap junctions and hemichannels that account for intercellular and extracellular syncytia, respectively [50,51]. Astrocytes regulate the extracellular environment, participate in synaptic activity, and promptly react to CNS lesions. They provide metabolic support to neurons, regulate the composition of the neuronal microenvironment, and regulate blood supply [52,53,54]. The regionality of astrocytes could partly explain the localization of neuropathological findings with various susceptibility of different brain areas [46]. In response to changes in synaptic activity, astrocytes release prostaglandins, arachidonic acid, and nitric oxide to constrict or vasodilate blood vessels. Vascular cerebral flow varies according to neuronal activity and energy demands [31,55]. The formation of arachidonic acid and the release of vasoactive substances are induced by changes in the level of intracellular calcium in astrocytes [56]. Thus, astrocyte dysfunction damages the BBB and induces alterations in the clearance of Aβ [57,58]. Alterations in tau protein and BBB dysfunction are also closely linked: just as tau pathology can trigger BBB damage, BBB dysfunction can induce tau hyperphosphorylation creating a deleterious feed-forward loop [59]. Damage to the BBB induces oxidative stress and neuroinflammation and thus can accelerate the development of tau hyperphosphorylation and NFT formation [60]. The BBB dysfunction could initiate tau pathology [61,62]. Preclinical studies have shown that the BBB can reversibly be opened by magnetic resonance (MR)-guided low-intensity focused ultrasound (FUS). This facilitates the delivery of targeted brain therapeutics. It has also been shown that FUS can safely, noninvasively, transiently, reproducibly, and focally mediate BBB opening in the hippocampus/EC in humans [63]. Different mouse models of Alzheimer’s disease have been used to investigate disease-specific changes in the BBB, but the translation of findings from mouse models into the human pathology is hindered by interspecies differences. Several human BBB in vitro models have been developed to create new delivery techniques of drugs into the brain, and to better understand the alterations of the BBB in the case of Alzheimer’s disease [64]. The homeostasis of protein production and clearance is not autonomously guaranteed by the brain, like it was thought, recycling its wastes [65]. The paradigm shift is due to the emerging role of sleep–wake cycles and the discovery of the related influx/efflux currents from the brain parenchyma to the lymphatic circulation, via astrocytes (the glymphatic system) [66]. These fluxes are generated by arterial pulsation which allows CSF influx from the perivascular space into the parenchyma, regulated by astrocytic end-feet which express clustered AQP4 water channels. The efflux of CSF mixed with extracellular interstitial fluid is directed towards the low-pressure venous system (perivenous spaces), where solutes are exported through meningeal lymphatic vessels [66]. During the sleep–wake cycle, these fluxes allow the variation of small molecules and protein concentrations (neurotransmitters, MMPs, etc.), change the localization of membrane receptors (e.g., LRP1, glutamate receptors), and rearrange the ECM complex structures bridging the cellular cytoskeletal elements [67,68]. Indeed, it has been shown that there is a link between single nucleotide polymorphisms (SNPs) of *AQP4* and the integrity of perivascular AQP4 localization, correlated to sleep quality and cognitive functions in humans [69,70]. Remarkably, *AQP4* is exclusively astrocytic and the expression of perivascular astroglial gene products, such as dystroglycan, dystrobrevin, and alpha-syntrophin, were also associated with dementia and phosphorylated tau levels in the temporal cortex [71]. The reduction of these fluxes could in part justify how sleep-related disorders and circadian rhythm dysfunctions are associated with AD [72]. The pathological blockage of the lymphatic flow at each point of the described pathway (e.g., decreased or dispersed *AQP4* expression, hydrostatic pressure variation due to CSF decline or inflammatory processes) could justify regionally specific degeneration, based on microvascular dynamics not utterly clarified [73]. Moreover, the hindrance to fluid passage from the neuropilum to the perivascular space together with the *LRP1* expression on smooth muscle cells surrounding the vessels could sequester Aβ, favoring vascular amyloidosis [66]. Eventually, the bottleneck of this system could be localized even outside the CNS in the cervical lymphatics [74,75]. Finally, the proteomics associated with the circadian rhythms showed that proteins involved in synaptic transmission were predominantly expressed during waking times, while metabolism genes were activated a few hours before the expected sleep. These characteristics were altered by sleep deprivation, with synaptic transmission overcoming the proteins functionally associated with metabolism. This emergency mechanism, if chronically activated, could lead to metabolic impairment and cognitive dysfunctions [76,77].

## 3. From Vascular Damage to Alteration of Metabolism

The role of NVU in AD should be emphasized as a whole. Whenever there is damage to the NVU, it can trigger the breakdown of the BBB, decrease cerebral blood flow, and decrease the clearance of Aβ and its deposition. The result is a response that alters the different elements of the NVU: pericyte degeneration, activation of glial cells, metabolic imbalance, activation of the neuroinflammatory response, synaptic impairment, and neuronal loss [78]. Following vascular damage, thrombin promotes the formation of fibrin and platelet aggregation. Fibrin, by causing an increase in inflammatory and oxidative mediators, activates glial cells and damages the blood–brain barrier (BBB) [79]. Thrombin, a multifunctional serine protease, is thought to be responsible for vascular dysfunction, inflammation, and neurodegeneration. It acts on the endothelial cells of the blood–brain barrier, on microglia, astrocytes, and neurons. In AD patients, its levels are elevated, and a correlation has been identified between thrombin signaling and pathological markers of the disease, tau protein, and beta-amyloid plaques. Understanding the effects of thrombin on BBB endothelial cells is crucial. On the one hand, the damage caused by thrombin increases the permeability of the BBB, allowing several harmful substances to enter the brain from the blood. On the other hand, the damaged endothelial cells themselves can produce thrombin, which has negative effects on astrocytes, microglia, and neurons [79]. Following vascular damage, a decrease in the clearance of Aβ and its deposition with significant metabolic alterations occurs. Regional changes in brain glucose metabolism seen during healthy ageing are quantitatively and qualitatively different from those in AD [80,81]. During healthy aging, some cognitive domains have a modest decline, such as episodic and working memory, whereas others (such as semantic memory) do not undergo significant changes [82]. In healthy aging, the frontal cortex is the region most affected by the reduction in brain glucose metabolism, whereas in AD, the parietal lobe and the precuneus are the most markedly affected [27,83,84,85,86]. APOE*E4 alleles (which encode the E4 isoform of apolipoprotein E (ApoE4)) carriers have reduced glucose metabolism [87] and increased accumulation of Aβ aggregates [27]. Region-specific neurodegenerative diseases (AD, PD, HD) appear to be caused by the accumulation of ROS in the injured regions, which result from the alteration of mitochondrial activity. Much data have accumulated to indicate that mitochondrial dysfunction, especially at the level of Complex I, accumulates with age, leading to the production of ROS and the reduced production of ATP building blocks, which prevents the necessary work for the maintenance of cells and organs. The synthesis of lipids at the neuronal level can be induced by the accumulation of high levels of reactive oxygen species (ROS) [88,89]. Peroxidized lipids are sequestered in the lipid droplets (LD) at the glial level, delaying neurotoxicity. The damaged mitochondria produce ROS, which in turn are responsible for activating the transcription factors *JNK* and *SREBP* which determine lipid synthesis. The synthesized lipids undergo the peroxidation process thanks to the presence of ROS. They are subsequently transferred to the pigment glia, where they are sequestered in the LDs [88]. Activation of neuronal lipogenesis, in the absence of ROS, determines the formation of LD but does not lead to neurodegeneration [90]. Therefore, lipid production, and not lipid peroxidation, causes neurotoxicity by damaging lipids, proteins, and nucleic acids [91,92]. ROS could have a beneficial role for cells when their levels are finely regulated [93,94]. Nowadays, it is not clear whether ROS are the cause or the consequence of the disease, but it is well known that their production is exacerbated by neuroinflammation [95,96] and by Aβ42-mediated neurotoxicity [91,97]. The formation of lipid droplets is therefore neuroprotective. Several genes regulate their formation, including homologues of human *ABCA1*, *ABCA7*, *VLDLR*, *VPS26*, *VPS35*, *AP2A*, *PICALM*, and *CD2AP*. *APOE* could be relevant as a risk factor for AD by mediating and modulating the transfer of lipids between neurons and the glia. Reduced transport capacities of *APOE4* [78,98,99] could be responsible for a reduced transfer of lipids between neurons and the glia. When ROS levels rise, the peroxidized lipids are no longer able to be exported and transferred to the glial cell LDs. This determines neuronal damage [88]. In a mouse model of human *APOE* expression [100,101], an ABCA1 agonist peptide was able to restore APOE4 lipidation and improve Aβ42/tau pathologies. However, a possible contribution to the formation of LDs has not been investigated. An ABCA1 agonist peptide was also studied in a humanized *APOE4* fly model. It has been shown to be able to restore glial LD formation. This could have a valid therapeutic potential for preventing ROS-induced neurotoxicity. In the future, it will be necessary to study therapeutic approaches aimed at inducing the glial uptake of lipids to reduce the levels of ROS and clear amyloids.

## 4. Glycometabolic Issues in Neurodegeneration: Brain Energy Rescue

Metabolism, through the production of ATP, regulates both the difference in electrochemical potential between mitochondria and the cell cytoplasm, and bioelectric activity, which propagates from the neuronal axons, and together they have a determining role in the transmission of the nerve impulse. Metabolism is considered as the core of destiny regulation, growth, death, or cell differentiation. Mathematical models are being developed to explain the connection between ROS and metabolism, as well as the importance of ROS-mitochondrial remodeling in neuron differentiation, neuroprotection, and antigliosis [53,102,103]. The brain continuously requires energy in the form of ATP. Although the brain accounts for just over 2% of an adult’s body weight, the brain demands 20% of the body’s total energy requirement. Glucose metabolism provides 95% or more of the brain’s production of ATP [104]. Most of it is produced by oxidative phosphorylation in the mitochondria starting from glucose, while another share is produced by aerobic glycolysis in the cytoplasm. The ATP required by neurons is mainly generated in the mitochondria, with the oxidative phosphorylation of glucose via the tricarboxylic acid cycle [105]. This is unlike astrocytes, which, on the other hand, satisfy their energy needs mainly through aerobic glycolysis [106]. When glucose decreases, the liver generates ketonic bodies and lactate, produced by skeletal muscles during exercise, and lactate is also used as an energy substrate [27]. The different cytotypes that together form the neurovascular unit (brain capillary endothelial cells, pericytes, astrocytes, oligodendrocytes, microglia, and neuron) manage glucose uptake within the brain [107,108]. Glucose uptake is not stimulated by its circulating levels but by the energy demands of activated neuronal cells [27]. Among the various glucose transporters involved, it is significant to mention GLUT4, whose translocation in the plasma membrane is insulin-dependent in muscles, adipose tissue, and probably also in neurons. This is why insulin resistance, which often occurs in NDAs, results in a reduction in neuronal glucose uptake [106,109]. Neuronal activation transiently stimulates aerobic glycolysis in astrocytes, thus producing lactate. According to the astrocyte-neuron lactate shuttle, the release of glutamate—mediated by neurons during neuronal transmission—stimulates glucose uptake, glycogen catabolism, aerobic glycolysis, and the production of lactate by surrounding astrocytes. Lactate produced by astrocytes is thought to contribute to neuroplasticity [27]. Ketone bodies and lactate are the main alternative energy sources to glucose. The two main ketone bodies are acetoacetate and D-beta-hydroxybutyrate (BHB). Only acetoacetate can be metabolized into acetyl coenzyme A (acetyl-CoA). The acetyl-CoA enters the TCA cycle to generate ATP. Unlike glucose uptake which varies according to neuronal metabolic activity, ketone entry is directly related to their plasma concentration [28]. This difference justifies the glucose-sparing effect of increased ketone levels [110]. Unlike glucose, ketone bodies can contribute to the production of ATP only through oxidative phosphorylation, not being able to exploit aerobic glycolysis and not being able to be metabolized to lactate [27]. Oligodendrocytes primarily obtain ATP through aerobic glycolysis, while microglia mainly exploit oxidative phosphorylation. In the case of NDAs, due to neuroinflammation, the microglia undergoes a metabolic reprogramming turning towards an aerobic glycolysis-predominant phenotype. In parallel with this energetic shift, microglia play a pathological role rather than a protective one against neurodegenerative diseases. When the energy resources of glucose are no longer sufficient, the high energy demand by the activated microglia further limits the energy available to neurons. The function of astrocytes and oligodendrocytes is perturbed, which leads to a worsening of the aging process which perpetuates and worsens neurodegeneration and cerebral glucose metabolism [27]. In neurodegenerative diseases, we witness a deterioration of the cerebral glucose metabolism in a progressive way and with a specific region distribution, depending on the pathology in question. Brain energy metabolism deteriorates over the course of aging and this decline is often present before the diagnosis of NDAs (neurodegenerative disorders of aging) [28,29]. Even before the diagnosis of AD is made, there is a disorder of glucose metabolism in some regions, but oxygen, lactate, and ketone levels do not undergo significant changes. Already in mild cognitive impairment (MCI) there is a deficit of glucose uptake of 10–12% in the entorhinal cortex and in the parietal lobes, a defect that becomes more and more accentuated as the disease progresses [27]. Reduced neuronal glucose uptake, impaired aerobic glycolysis and TCA cycle, failure of axonal transport, and loss of glial energy support to neurons are major causes of cerebral glucose hypometabolism in NDAs. Lower glucose uptake, TCA activity, mitochondrial function, and energy support of neuronal astrocytes and oligodendrocytes characterize AD [27]. Neuronal network performance and axonal mitochondrial transport are impaired by white matter atrophy in AD. Particularly in women, the loss of white matter is due to the reduction of maintenance and synthesis of myelin (energy-intensive processes) and the catabolism of myelin to provide energy in the occurrence of a reduction in glucose levels [111]. Brain glucose hypometabolism is associated with synaptic loss and neuronal death in AD. This is followed by the onset of energy deficits and the accumulation of neurotoxic proteins which further worsen the situation. The clearance of the 42 amino acid isoform of beta-amyloid proteins (Aβ42) and tau from the brain is impaired by an insufficient generation of neuronal glucose and mitochondrial energy. In turn, the accumulation of Aβ42 and tau worsens mitochondrial homeostasis and energy production; this process is based on an increase in oxidative stress [112,113]. Aβ exacerbates cerebral glucose hypometabolism, possibly due to pericyte-mediated constriction of capillary blood flow. In turn, this hypometabolism triggers cellular damage and neuroinflammation [113,114]. Furthermore, the mitophagic process becomes insufficient, resulting in an accumulation of impaired mitochondria that further compromise bioenergetics in AD [115]. The metabolism of brain ketones, unlike the use of brain glucose, is not altered in Alzheimer’s disease (AD) and mild cognitive impairment (MCI), which implies that mitochondrial respiration is relatively normal in most mitochondria; this allows the generation of ATP. So, understanding if the metabolism of brain ketones is compromised allows us to go and study the possible alteration of mitochondrial activity. In fact, if the dysfunction is only a burden of glycolysis or glucose transport, it means that only glucose metabolism is compromised. Ketogenic food supplements containing medium-chain triglycerides and the very low-carb ketogenic diet improve cognitive function in AD, as has been shown in several clinical studies [27]. This deterioration opens up new therapeutic perspectives to address neurodegenerative diseases; the conditions of these patients could be improved and slowed down by preserving or rescuing brain energetics. The possible approaches are numerous: it could restore the functionality of oxidative phosphorylation and glycolysis, it could increase insulin sensitivity, correct mitochondrial dysfunction, perform ketone-based interventions, act through hormones to modulate brain energy, RNA therapeutics, and aid in lifestyle changes. Impaired brain energy metabolism precedes the onset of the NDA clinic. There is a reduction in neuronal glucose uptake, as well as alterations in glycolysis and in the functioning of the TCA cycle, with further negative consequences on mitochondrial function and the production of ATP. Now, we need to consider a combined approach that includes both the use of drugs and improvements in diet and lifestyle to improve the prognosis and clinical symptoms of patients with neurodegenerative diseases [27]. Lifestyle changes that rely primarily on increasing physical activity and improving one’s diet can reduce insulin resistance and improve brain energy, and thus reduce the occurrence of neurodegenerative diseases. Both increased physical activity and improved diet are neuroprotective in preclinical models of AD [116,117,118,119]. They cause increased neurogenesis in the hippocampus, increased autophagy of neurotoxic proteins, and increased mitophagy by removing impaired mitochondria [82,120,121] and biogenesis of increased synaptic spine density [122].

## 5. Conclusions and Perspectives

In conclusion, we can understand how saving brain energy improves neuronal integrity, synaptic plasticity, and interactions between neurons and glia, delaying the onset and progression of neurodegenerative diseases. Just as the neurocognitive development of the child requires an adequate energy supply, in the same way this energy supply is necessary during aging. To delay the onset and progression of neurodegenerative diseases, maintaining the brain’s energy state should therefore be a cornerstone for future experiments. Diet, body weight, diabetes, physical activity, and other factors must be kept under control. We cannot cure dementia, but we do know how to decrease its risk. It is increasingly clear that it is not monocausal but a multi-cause syndrome. This changes the conceptual and practical approach to dementia: preventive measures are exploited, which act on the epigenesis of dementia. Now we can talk of the genetic and epigenetic nature of senile dementia. Metabolic connectivity is the key word for the future study of neurodegenerative diseases. It means the intertwining of the metabolic pathways for which the increase in the level of a certain metabolite may correspond to the increase (or the decrease) of other metabolites, depending on the metabolic pathway taken. Each disturbance of cell physiology will be characterized by having its own “metabolic fingerprint”, i.e., variations of metabolite levels in certain areas of metabolism, which will be strictly specific with respect to the physiological disturbance. Given the great level of connectivity of the metabolism, it will be difficult to analyze and interpret these metabolic changes without the use of mathematical models. According to Systems Biology, therefore, the functional properties of the cell result in emerging properties, directly determined by metabolism. Understanding how metabolism is regulated and how all other events occur, as well as how informational cells act on metabolism, and how metabolites act on other events of cellular life, is far from simple. A mosaic is not examined by concentrating attention on the individual tiles: the most detailed analysis of the parts cannot in fact provide an idea of the whole. Numerous future studies are needed to delve into this new fundamental view based on Systems Biology and metabolism. These approaches which will constitute the “systems metabolomics” studies will be the new cornerstones for the interpretation of biological processes.
